# Safety and Efficacy of Pelvic Reinforcement Procedure for Preventing Postoperative Perineal Hernia After Robotic Abdominoperineal Resection: A Single‐Center, Retrospective Cohort Study

**DOI:** 10.1002/ags3.70066

**Published:** 2025-07-15

**Authors:** Yoshihiro Sakai, Shunsuke Kasai, Akio Shiomi, Shoichi Manabe, Yusuke Yamaoka, Yusuke Tanaka, Takahiro Igaki, Hiroyasu Kagawa, Yusuke Kinugasa

**Affiliations:** ^1^ Division of Colon and Rectal Surgery Shizuoka Cancer Center Shizuoka Japan; ^2^ Department of Gastrointestinal Surgery Institute of Science Tokyo Tokyo Japan

**Keywords:** abdominoperineal resection, pelvic reinforcement procedure, perineal hernia, rectal cancer, robotic surgery

## Abstract

**Aim:**

Few reports have described pelvic reinforcement procedure (PRP) to prevent perineal hernia (PH) in robotic abdominoperineal resection (Ro‐APR) for rectal cancer. This study aimed to investigate the safety and efficacy of PRP in Ro‐APR.

**Methods:**

Patients who underwent Ro‐APR for rectal cancer between January 2020 and June 2023 were retrospectively examined. PRP was performed as a prophylactic procedure for PH. Four types of PRP were performed depending on the case (closure of the levator ani muscles, the pelvic peritoneum with the uterus, the pelvic peritoneum, and the pelvic peritoneum with a bladder peritoneal flap). Background factors and surgical outcomes were compared between patients without PRP (PRP−) and with PRP (PRP+). Imaged PH was diagnosed using computed tomography 1 year postoperatively. Imaged PH with symptoms was defined as symptomatic PH.

**Results:**

We evaluated 81 patients, including 51 PRP− (63.0%) and 30 PRP+ (37.0%). There were no differences in the characteristics between the two groups. There was no significant difference in operative time between the two groups (358 min vs. 329 min, *p* = 0.460). PRP− had a significantly higher rate of imaged PH (39.2% vs. 6.7%, *p* = 0.005) and symptomatic PH (19.6% vs. 3.3%, *p* = 0.047). The two groups had no significant differences in the other postoperative complications. In multivariate analysis, the independent risk factor for PH was not undergoing PRP (odds ratio 9.71, *p* = 0.005).

**Conclusion:**

PRP in Ro‐APR for rectal cancer can be safely performed and helps prevent PH.

## Graphical abstract

This study aimed to investigate the safety and efficacy of pelvic reinforcement procedure (PRP) in robotic abdominoperineal resection (Ro‐APR). PRP‐ had a significantly higher rate of imaged perineal hernia (PH) (39.2% vs. 6.7%, *p* = 0.005) and symptomatic PH (19.6% vs. 3.3%, *p* = 0.047), and there were no significant differences in the other postoperative complications between PRP‐ and PRP+. PRP in Ro‐APR for rectal cancer can be safely performed and helps prevent PH.

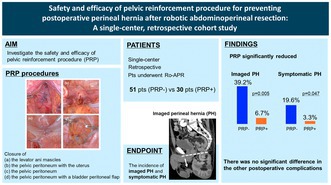

## 1 Introduction

Surgery is the gold standard of treatment for rectal cancer [1]. In particular, resection of low rectal cancer is critical for reliable local control but is technically demanding in the narrow and deep pelvis. In recent years, the number of patients undergoing super‐low anterior resection or intersphincteric resection for low rectal cancer has increased [2, 3], enabling preservation of the anus while ensuring oncological radicality in many cases. However, there are still cases in which abdominoperineal resection (APR) remains an absolute indication for cancer treatment. APR is indicated for patients with low rectal cancer, particularly those with sphincter invasion, large tumors, and poor sphincter function.

APR requires a perineal procedure without anastomosis, which may lead to specific complications and, therefore, requires careful attention. With the increasing adoption of minimally invasive surgery (MIS) for rectal cancer, the advantages of robotic APR (Ro‐APR) in preventing complications have been reported [4]. However, postoperative perineal hernia (PH) has been increasing to approximately 15%–40% with the increasing use of MIS [5, 6, 7]. The increase in PH was considered to be caused by a reduction of adhesions in MIS. PH can cause discomfort and pain associated with perineal bulging and may lead to bowel obstruction, potentially reducing the patient's quality of life [8, 9]. Therefore, we consider PH a significant complication that should be prevented in Ro‐APR.

Pelvic floor reconstruction using mesh or myocutaneous flaps has been performed to prevent PH; however, these methods are complex procedures with high costs and are time‐consuming [10, 11]. In recent years, the usefulness of pelvic reinforcement procedure (PRP), a more straightforward method without mesh or myocutaneous flaps for preventing PH in APR, has been suggested [7, 12]. PRP in Ro‐APR has also been suggested to be effective in preventing PH [13]. The complex surgical techniques involved in PRP, including suturing, may be performed more easily with robotic surgery than with conventional laparoscopic surgery. However, the safety and efficacy of robotic PRP have not been thoroughly investigated. This study describes the robotic PRP technique and evaluates its safety and efficacy.

## 2 Method

### 2.1 Patient Selection and Definition of PH

We retrospectively examined the data of patients who underwent Ro‐APR for rectal cancer between April 2020 and October 2023 at Shizuoka Cancer Center Hospital, a high‐volume center in Japan. Patients who had a history of treatment for pelvic malignancies, had undergone surgery for local recurrence, had perineal closure with a myocutaneous flap performed by plastic surgeons, or were followed up for less than 1 year after surgery were excluded. Due to the retrospective nature of the study, written informed consent was not obtained. Regarding the use of patient data for research purposes, we used an opt‐out approach to disclose the study information. This consent procedure was reviewed and approved by the Institutional Review Board of Shizuoka Cancer Center (institutional code: J2024‐45–2024‐1–3).

**FIGURE 1:**
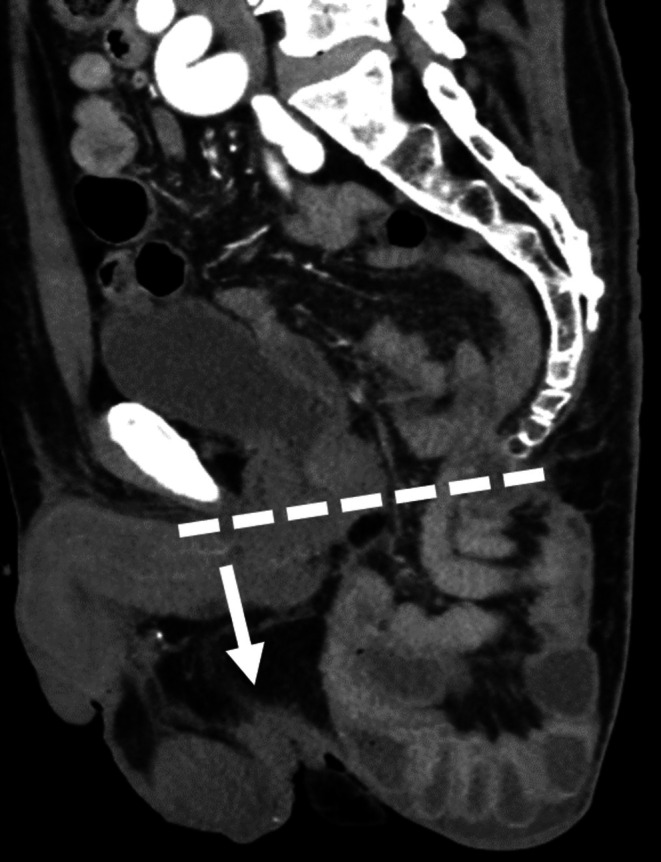
Definition of perineal hernia. Perineal hernia was defined as the downward displacement of the intestine beyond the line drawn by computed tomography from the inferior margin of the pubis to the edge of the coccyx, as previously reported.

We evaluated the imaged PH, defined as the downward displacement of the intestine beyond the line drawn by computed tomography from the inferior margin of the pubis to the edge of the coccyx, as previously reported [14] (Figure 1). Computed tomography 1 year after surgery was retrospectively evaluated. Imaged PH with symptoms was defined as symptomatic PH. We evaluated the symptoms of PH (pain, discomfort, and other symptoms) in the postoperative outpatient clinic by interviewing the patients.

### 2.2 Treatment Strategies for Low Rectal Cancer

In our department, patients diagnosed with rectal cancer typically undergo upfront robotic surgery without preoperative treatments [15]. APR is recommended for patients with low rectal cancer invading the levator ani or external anal sphincter muscle and for patients with tumors with unclear anal side margins, such as type 3 tumors and poorly differentiated adenocarcinomas [4]. In addition, APR is considered suitable for patients with impaired anorectal function.

Lateral lymph node dissection (LLND) was performed according to the Japanese Society for Cancer of the Colon and Rectum guidelines for rectal cancer surgery [16]. LLND was not performed in patients aged ≥ 75 years or those with severe comorbidities; however, unilateral LLND was considered a metastatic site [17]. In addition, preoperative chemoradiotherapy (CRT) (50.4 Gy in 28 fractions for 6 weeks with systemic capecitabine chemotherapy) was indicated only in patients requiring tumor shrinkage to obtain clear resection margins [18]. The preoperative diagnosis and treatment strategies were determined through multidisciplinary team discussions.

### 2.3 PRP Procedures

**FIGURE 2:**
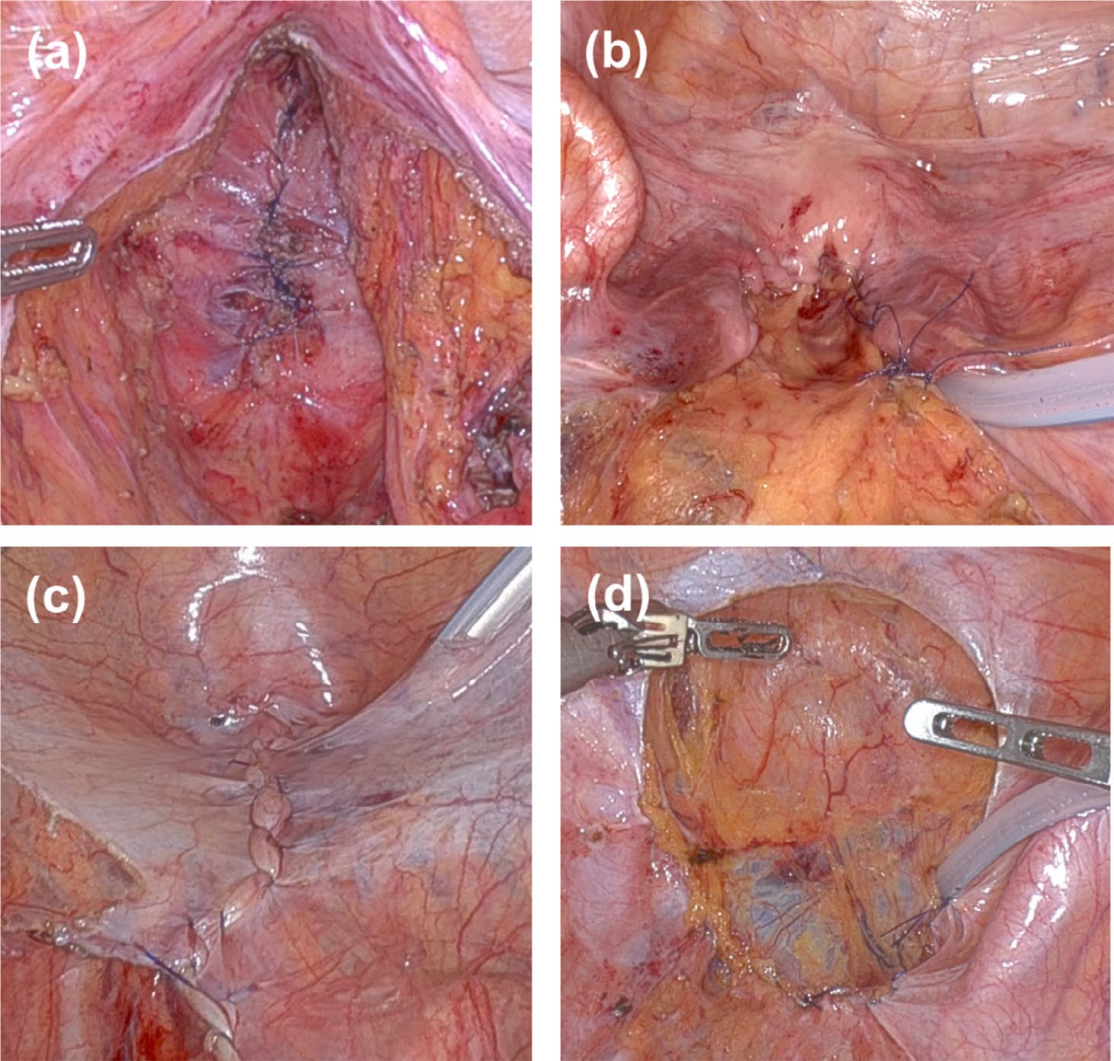
Pelvic reinforcement procedure. (a) Closure of the levator ani muscles. (b) Closure of the pelvic peritoneum with the uterus. (c) Closure of the pelvic peritoneum. (d) Closure of the pelvic peritoneum with a bladder peritoneal flap.

All patients underwent Ro‐APR using the principle of total mesorectal excision. The surgical procedure was performed as described in a previous article [4]. Perineal wounds are typically closed using primary closure. In addition, PRP has been performed as a prophylactic procedure for PH. In the following procedures, we used an absorbable monofilament yarn with fixating barbs as a suturing device, except for the closure of the levator ani muscles in the perineal procedure. Details of the surgical techniques for the closure of the levator ani muscles (Figure 2a), pelvic peritoneum with the uterus (Figure 2b), and pelvic peritoneum (Figure 2c) have been previously reported [13]. In addition to these methods, the closure of the pelvic peritoneum with a bladder peritoneal flap (Figure 2d) was performed if the closure of the pelvic peritoneum was difficult in male patients who underwent preoperative CRT or in those who underwent LLND [19]. In all patients who underwent the closure of the pelvic peritoneum with a bladder peritoneal flap, anti‐adhesion agents were applied to both the dissected surface of the bladder and the surface of the bladder peritoneum flap to prevent postoperative adhesions.

PRP (closure of the levator ani muscles, pelvic peritoneum with the uterus, and pelvic peritoneum) was introduced in April 2020 in our department. Initially, it was an exploratory surgical technique performed by a limited number of surgeons on selected cases. The number of cases in which PRP was performed increased after confirming its usefulness [13]. Furthermore, since August 2023, the use of the closure of the pelvic peritoneum with a bladder peritoneal flap has made it possible to perform the procedure even in patients who underwent preoperative CRT or in those who underwent LLND. Currently, PRP is performed in almost all Ro‐APR cases.

### 2.4 Outcome Measures and Statistical Analyses

We examined the safety and efficacy of PRP in Ro‐APR, compared the characteristics between patients without PRP (PRP−) and those with PRP (PRP+), and identified the risk factors for PH. Postoperative complications occurring within 30 days following surgery were assessed according to the Clavien–Dindo classification system [20]. Fisher's exact test was used to evaluate categorical variables, while the Mann–Whitney *U* test was used to compare continuous variables between groups. Logistic regression analysis was conducted to identify risk factors for PH. Variables with *p*‐values < 0.1 in univariate analysis were incorporated into the multivariate analysis model. Statistical significance was set at a two‐sided *p*‐value < 0.05. All analyses were performed using EZR software (Saitama Medical Center, Jichi Medical University, Saitama, Japan) [21].

## 3 Results

**FIGURE 3:**
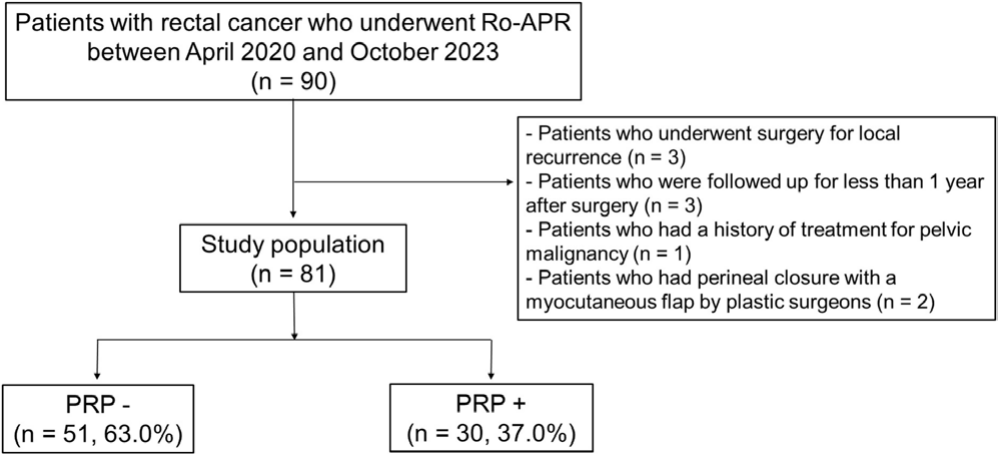
Patient selection. PRP−, patients without pelvic reinforcement procedure; PRP+, patients with pelvic reinforcement procedure; Ro‐APR, robotic abdominoperineal resection.

During the study period, Ro‐APR was performed in 90 patients with rectal cancer. We excluded three patients who underwent surgery for local recurrence, three patients who were followed up for less than 1 year after surgery, one patient who had a history of treatment for pelvic malignancies, and two patients who underwent perineal closure with a myocutaneous flap by plastic surgeons. Consequently, we evaluated 81 patients, including 51 PRP− (63.0%) and 30 PRP+ (37.0%) (Figure 3).

Table 1 lists the characteristics of the PRP− and PRP+ cells. There was no difference in preoperative diagnosis between the two groups, including preoperative CRT (13.7% vs. 6.7%, *p* = 0.473).

**TABLE 1:** Patient characteristics.

	PRP−	PRP+	*p*
	(*n* = 51)	(*n* = 30)	
Age (year)	75 [45–90]	73.5 [46–90]	0.449
Sex			
Male	26 (51.0)	17 (56.7)	0.777
Female	25 (49.0)	13 (43.3)	
BMI (kg/m^2^)	22.9 [14.4–40.7]	21.5 [15.2–30.7]	0.591
ASA classification			
I	9 (17.6)	4 (13.3)	0.814
II	35 (68.6)	23 (76.7)	
III	7 (13.8)	3 (10.0)	
Preoperative chemoradiotherapy	7 (13.7)	2 (6.7)	0.473
Tumor distance from anal verge (cm)	2.5 [0–7]	2.75 [0–7]	0.713
Tumor size	5.0 [2.0–10.0]	4.5 [1.2–8.0]	0.081
Histological type			
Pap/tub1/tub2	47 (92.2)	25 (83.3)	0.345
Por/sig/muc	4 (7.8)	3 (10.0)	
Neuroendocrine tumor	0 (0)	1 (3.3)	
Paget disease	0 (0)	1 (3.3)	
c/yc T			
0	0 (0)	1 (3.3)	0.348
1	3 (5.9)	2 (6.7)	
2	3 (5.9)	5 (16.7)	
3	31 (60.8)	15 (50.0)	
4	14 (27.4)	7 (23.3)	
c/yc N			
0	26 (51.0)	14 (46.7)	0.824
1	15 (29.4)	11 (36.6)	
2	10 (19.6)	5 (16.7)	
c/yc M			
0	48 (94.1)	28 (93.3)	1.000
1	3 (5.9)	2 (6.7)	

*Note:* Values are indicated as frequencies (percentages) or medians [ranges].

Abbreviations: ASA, American Society of Anesthesiology; BMI, body mass index; muc, mucinous adenocarcinoma; Pap, papillary adenocarcinoma; por, poorly differentiated adenocarcinoma; PRP+, patients with pelvic reinforcement procedure; PRP−, patients without pelvic reinforcement procedure; tub1, well‐differentiated tubular adenocarcinoma; tub2, moderately differentiated tubular adenocarcinoma.

Table 2 shows operative outcomes. Operative time was insignificant between the two groups (358 min vs. 329 min, *p* = 0.460). The amount of blood loss was significantly higher in PRP− (53 mL vs. 27 mL, *p* = 0.001). There was no significant difference in LLND rate between the two groups. PRP− had a significantly higher rate of imaged PH (39.2% vs. 6.7%, *p* = 0.005) and symptomatic PH (19.6% vs. 3.3%, *p* = 0.047). As for postoperative complications (Clavien‐Dindo classification ≥ grade II), including perineal wound infection and urinary disorder, there was no significant difference between the two groups (33.3% vs. 36.7%, *p* = 0.812).

**TABLE 2:** Operative outcomes.

	PRP−	PRP+	*p*
	(*n* = 51)	(*n* = 30)	
Operative time (min)	358 [143–676]	329 [206–643]	0.460
Blood loss (mL)	53 [0–705]	27 [0–811]	0.001
Resection of adjacent organs	20 (39.2)	11 (26.7)	1.000
Seminal vesicle	2 (3.9%)	2 (6.7%)	0.624
Prostate	3 (5.9%)	3 (10.0%)	0.665
Uterus	3 (5.9%)	0 (0%)	0.292
Vagina	15 (29.4%)	7 (23.3)	0.613
Lateral lymph node dissection			
Absent	27 (52.9)	17 (56.7)	0.953
Unilateral	10 (19.6)	5 (16.7)	
Bilateral	14 (27.5)	8 (26.6)	
Anti‐adhesion agents	5 (9.8)	8 (26.7)	0.062
Complication (CD ≥ grade II)			
Total patient number	17 (33.3)	11 (36.7)	0.812
Perinial wound infection	11 (21.6)	3 (10)	0.234
Small bowel obstruction	1 (2.0)	0 (0)	1.000
Bleeding	1 (2.0)	2 (6.7)	0.552
Urinary dysfunction^a^	7 (13.7)	3 (10)	0.739
Others	2 (3.9)	4 (13.3)	0.187
Imaged perineal hernia	20 (39.2)	2 (6.7)	0.005
Symptomatic perineal hernia	10 (19.6)	1 (3.3)	0.047
Postoperative hospital stays (days)	7 [6–39]	6 [6–20]	0.082
Readmission	3 (5.9)	4 (13.3)	0.414

*Note:* Values are presented as the number (percentage) or median [range].

Abbreviations: CD, Clavien–Dindo classification; PRP−, patients without pelvic reinforcement procedure; PRP+, patients with pelvic reinforcement procedure.

^a^ Urinary dysfunction was defined as the presence of > 50 mL of residual urine volume, excluding the presence of urinary dysfunction before surgery.

Table 3 shows the details of the PRP. All patients who underwent closure of the pelvic peritoneum and those who underwent closure with a bladder peritoneal flap were male. Three patients who underwent preoperative CRT were performed with only closure with a bladder peritoneal flap. Except for the closure of the levator ani muscles, closure with a bladder peritoneum flap tended to have a longer procedure time than the closure of the pelvic peritoneum and the uterus. Anti‐adhesion agents were used for all patients who underwent closure with a bladder peritoneum flap. Patients who underwent PRP did not develop imaged PH, except for one who underwent closure of the pelvic peritoneum and one who underwent closure with the uterus. Moreover, symptomatic PH occurred in one patient who underwent closure of the pelvic peritoneum.

**TABLE 3:** Details of pelvic reinforcement procedure.

	Levator ani muscle^a^	Pelvic peritoneum with uterus	Pelvic peritoneum	Pelvic peritoneum with bladder peritoneal flap
	(*n* = 4)	(*n* = 11)	(*n* = 9)	(*n* = 6)
Sex				
Male	2 (50.0)	0 (0)	9 (100.0)	6 (100.0)
Female	2 (50.0)	11 (100.0)	0 (0)	0 (0)
Preoperative chemoradiotherapy	0 (0)	0 (0)	0 (0)	2 (33.3)
Operative time (min)	353 [252–504]	304 [255–454]	329 [242–452]	413 [206–643]
Closure time (min)	33^a^	17 [15–30]	20 [8–27]	28 [19–30]
Blood loss (mL)	21 [0–58]	21 [0–55]	5 [0–200]	82 [0–811]
Lateral lymph node dissection				
Absent	1 (25.0)	7 (63.6)	5 (55.6)	4 (66.7)
Unilateral	2 (50.0)	1 (9.1)	2 (22.2)	0 (0)
Bilateral	1 (25.0)	3 (27.3)	2 (22.2)	2 (33.3)
Anti‐adhesion agents	1 (25.0)	0 (0)	1 (11.1)	6 (100)
Imaged perineal hernia	0 (0)	1 (9.1)	1 (11.1)	0 (0)
Symptomatic perineal hernia	0 (0)	0 (0)	1 (11.1)	0 (0)

*Note:* Values are indicated as frequencies (percentages) or medians [ranges].

^a^ Three patients underwent suturing of the levator ani muscles in the perineal procedure, and one patient underwent robotic closure of the levator ani muscles whose procedure time was 33 min.

Table 4 presents the risk factors for imaged PH. In univariate analysis, the significant risk factors were not undergoing LLND (odds ratio [OR] 2.950, 95% confidence interval [CI] 1.010–8.590, *p* = 0.047) and not undergoing PRP (OR 9.009, 95% CI, 1.930–42.200; *p* = 0.005). In the multivariate analysis, the independent risk factor for imaged PH was not undergoing PRP (OR 9.709, 95% CI, 2.000–47.000; *p* = 0.005).

**TABLE 4:** Univariate and multivariate analyses of risk factors for perineal hernia.

	Univariate analysis		Multivariate analysis	
	OR (95% CI)	*p*	OR (95% CI)	*p*
Age ≥ 75 (vs. < 75) (year)	2.740 (0.994–7.550)	0.051	1.820 (0.581–5.700)	0.304
Sex male (vs. female)	1.290 (0.472–3.520)	0.620		
BMI ≥ 25.0 (vs. < 25.0) (kg/m^2^)	1.500 (0.510–4.410)	0.461		
Preoperative chemoradiotherapy (vs. absent)	2.400 (0.581–9.920)	0.227		
Tumor size ≥ 5.0 (vs. < 5.0) (cm)	1.400 (0.518–3.760)	0.509		
cT ≥ 4 (vs. cT ≤ 3)	1.500 (0.510–4.410)	0.461		
Resection of adjacent organs (vs. absent)	1.510 (0.558–4.080)	0.418		
Anti‐adhesion agents− (vs. +)	2.290 (0.465–11.300)	0.308		
LLND− (vs. LLND+)	2.950 (1.010–8.590)	0.047	3.140 (0.956–10.300)	0.059
PRP− (vs. PRP+)	9.009 (1.930–42.200)	0.005	9.750 (2.000–47.000)	0.005

Abbreviations: BMI, body mass index; CI, confidence interval; LLND, lateral lymph node dissection; OR, odds ratio; PRP−, patients without pelvic reinforcement procedure; PRP+, patients with pelvic reinforcement procedure.

## 4 Discussion

In this retrospective study, PRP was a safe procedure, enabling pelvic floor reconstruction in patients with low rectal cancer undergoing Ro‐APR without increasing the incidence of intraoperative and postoperative complications. Furthermore, PRP reduced the incidence of imaged and symptomatic PH after Ro‐APR, and not undergoing PRP was an independent risk factor for imaged PH. To the best of our knowledge, this study is the first to comprehensively investigate PRP in Ro‐APR.

Previous reports on the incidence of PH in laparoscopic APR (Lap‐APR) indicated that the rate of imaged PH is approximately 15%–40% in cases where PRP was not performed, and PH occurs at a higher rate than open surgery [5, 6, 7]. In this study, the incidence of imaged PH was 39.2% in cases without PRP, consistent with previously reported rates of PH in Lap‐APR. The usefulness of PRP in preventing PH in Lap‐APR has been reported, with an incidence of imaged PH of 5%–10% in cases where PRP was performed [6, 7]. In previous reports, no imaged PH has been observed in cases where the pelvic floor was closed using a bladder peritoneal flap in Lap‐APR [19, 22]. In this study, the incidence of imaged PH in patients with PRP was similar, and no imaged PH was observed in patients who used a bladder peritoneal flap for PRP. Some reports suggested that acquiring suturing techniques was easier in robotic surgery than in laparoscopic surgery [23]. Therefore, PRP is expected to be more widely adopted in Ro‐APR in the future.

Previous studies identified several potential risk factors for PH after APR. These included smoking, a history of hysterectomy, a wide female pelvis, excessive length of the small intestinal mesentery, coccygectomy, and preoperative CRT, which were identified as substantial risk factors [24]. A report from our institution also identified the absence of PRP, preoperative CRT, and the absence of LLND as risk factors for PH after Ro‐APR [13]. In this study, the absence of PRP was the only independent risk factor. The reduction in PH of preoperative CRT‐treated patients who underwent PRP using a bladder peritoneal flap may explain why preoperative CRT was not identified as a risk factor in this study. Although the absence of LLND was not an independent risk factor for PH, it was a significant risk factor in the univariate analysis. Patients who underwent LLND tended to have a lower incidence of PH because a wide dissection of the lateral pelvis could lead to widespread adhesions in the pelvis and prevent the small intestine from falling into the center of the pelvis.

Various pelvic floor reconstructions, other than those used in this study, have been performed to prevent PH. Mesh reconstruction, a commonly used prophylactic method in APR, ensures effective pelvic floor reconstruction. It has been reported to reduce symptomatic PH but does not improve patients' quality of life [10]. Additionally, it is expensive and associated with a higher risk of potential complications such as internal hernias or infections [11, 25]. Myocutaneous flap reconstruction is an effective option to improve perineal wound healing. This method reduces the risk of PH after APR, although it can increase the operative time and risk of specific complications [26]. In contrast, PRP methods in this study demonstrated effectiveness in preventing PH and were easy, safe, and cost‐efficient without increasing intraoperative and postoperative complications.

**FIGURE 4:**
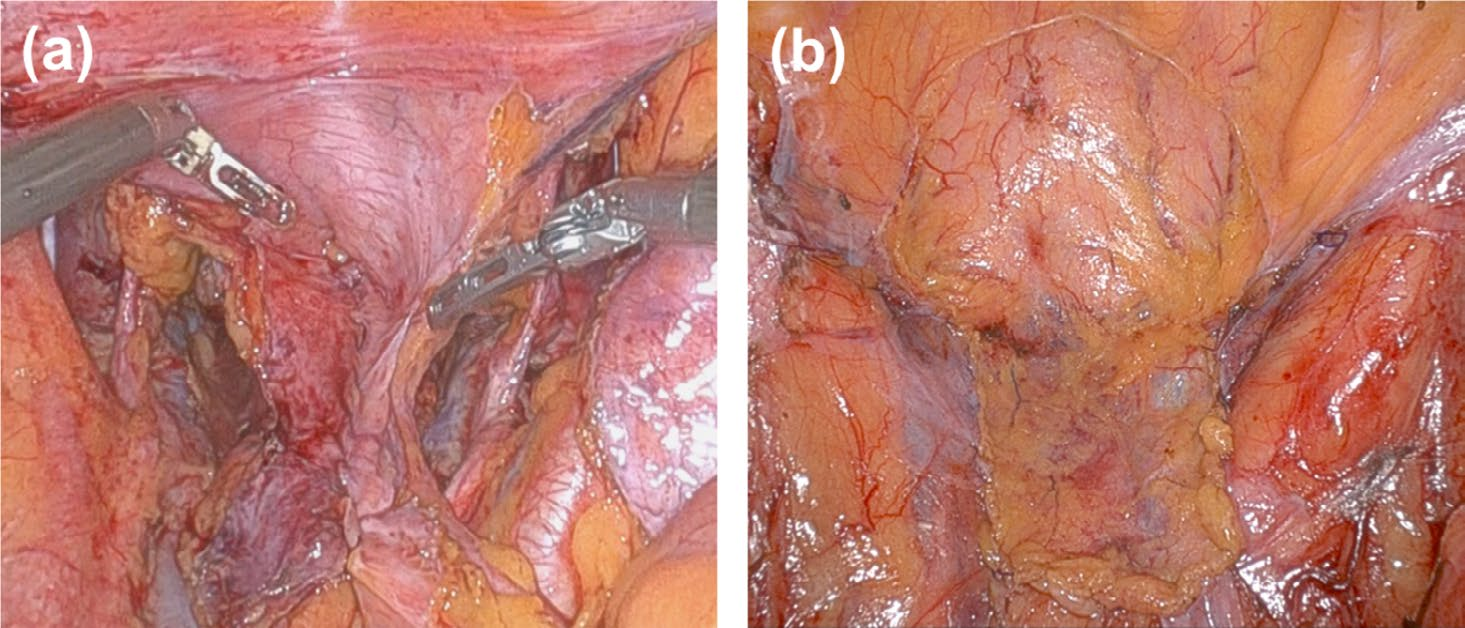
Pelvic reinforcement procedure for patients who underwent lateral lymph node dissection. Closure of pelvic peritoneum. Closure of the pelvic peritoneum with a bladder peritoneal flap.

Among the four PRP methods performed in our department, closure of the pelvic peritoneum with a bladder peritoneal flap has garnered significant attention in recent years [19, 22]. This technique was beneficial for male patients with a history of preoperative radiation therapy to the pelvis. Following radiotherapy, the pelvic peritoneum becomes fibrotic, making simple suture closure challenging. Yang et al. reported the usefulness of PRP using a bladder peritoneal flap after preoperative CRT in Lap‐APR at first [19], and the safety and utility of PRP using a bladder peritoneal flap in patients undergoing preoperative CRT have been demonstrated in a prospective phase II trial by Shen et al. [22]. Additionally, patients who have undergone LLND often present with extensive pelvic peritoneal defects. While simple suture closure could seal the central pelvic area, it was difficult to close the lateral cavity entrance simultaneously, potentially resulting in adhesions in those areas (Figure 4a). In contrast, PRP using a bladder peritoneal flap can cover pelvic peritoneal defects including the lateral cavity entrance, making it useful not only for preventing PH but also for reducing the risk of adhesion in the deep lateral pelvic cavity (Figure 4b). To our knowledge, this is the first study to investigate the safety and efficacy of PRP with a bladder peritoneal flap in patients undergoing LLND. Preoperative CRT and LLND are essential treatment strategies for local control of rectal cancer [27, 28]. Therefore, PRP using a bladder peritoneal flap may be important in preventing PH after APR with preoperative CRT and LLND. Furthermore, in Western countries, total neoadjuvant therapy (TNT), which includes systemic chemotherapy as a preoperative treatment before or after CRT, has recently been presented as the preferred approach for rectal cancer [29]. PRP using a bladder perineal flap for patients undergoing APR after TNT is considered useful for preventing PH. However, prolonged treatment duration before surgery has been reported to increase the difficulty of surgical procedures [30], indicating the necessity for additional research on the safety and efficacy of PRP in patients undergoing APR after TNT.

This study has some limitations. First, it was a retrospective cohort study conducted at a single institution. Although this study included patients who underwent preoperative CRT, LLND, and a novel approach to PRP, a similar study that included many patients at multiple institutions is necessary. In addition, PRP was initially an exploratory surgical technique performed by a limited number of surgeons on selected cases; therefore, this study may be subject to selection bias. A well‐designed prospective randomized controlled study is required to examine the effectiveness of PRP. Second, PH was evaluated using only CT images at 1 year, since previous studies revealed that most patients developed PH within 1 year after surgery [10, 14]; however, PH can occur after 1 year. A longer follow‐up time is needed, and complications other than PH require long‐term follow‐up. Particularly, the possibility should be noted that the peritoneum was not an adequate tissue to provide strength to the pelvic floor, so patients who underwent closure of peritoneum may develop PH in the long‐term follow‐up. Furthermore, in this study, four different PRP procedures were collectively categorized as PRP+; therefore, further accumulation of cases for each PRP subtype and longer follow‐up periods are needed. Finally, we identified the absence of PRP as a risk factor for PH. Owing to the small number of cases and retrospective design, it remained unknown whether there were other risk factors. In this study, the number of patients who underwent preoperative CRT was small, which may have limited our ability to accurately evaluate preoperative CRT as a risk factor.

In conclusion, PRP in Ro‐APR for rectal cancer can be safely performed and helps prevent PH without increasing intraoperative and postoperative complications. Although the evolution of oncological treatments for rectal cancer in recent years has been remarkable, further studies are needed to prevent complications and improve the patient's quality of life after surgery. We should continue to explore the unique risk factors of postoperative complications and develop innovative prevention methods.

## Author Contributions

**Yoshihiro Sakai:** conceptualization, methodology, writing – original draft, data curation, investigation, formal analysis, visualization. **Shunsuke Kasai:** conceptualization, investigation, writing – review and editing, supervision. **Akio Shiomi:** writing – review and editing, supervision. **Shoichi Manabe:** writing – review and editing. **Yusuke Yamaoka:** writing – review and editing. **Yusuke Tanaka:** writing – review and editing. **Takahiro Igaki:** writing – review and editing. **Hiroyasu Kagawa:** writing – review and editing. **Yusuke Kinugasa:** writing – review and editing, supervision.

## Ethics Statement

All study protocols were approved by the Institutional Review Board of the Shizuoka Cancer Center (institutional code: J2024‐45–2024‐1–3). Information about the study was disclosed on the Shizuoka Cancer Center website to ensure participants could opt out.

## Conflicts of Interest

Y.K. is an Associate Editor of Annals of Gastroenterological Surgery, and received lecture fees from Johnson and Johnson, Intuitive Surgical. The other authors declare no conflicts of interest.
